# 基于下一代测序分析*ROS1*阳性非小细胞肺癌的临床病理特征

**DOI:** 10.3779/j.issn.1009-3419.2026.106.13

**Published:** 2026-05-20

**Authors:** Lina GAO, Li SUN, Weihua LI, Jiali MU, Jinduo QIAO, Lei GUO

**Affiliations:** 100021 北京，国家癌症中心，国家肿瘤临床医学研究中心，北京协和医学院/中国医学科学院肿瘤医院病理科; Department of Pathology, National Cancer Center/National Clinical Research Center for Cancer/Cancer Hospital,Chinese Academy of Medical Sciences and Peking Union Medical College, Beijing 100021, China

**Keywords:** 肺肿瘤, *ROS1*基因融合, 下一代测序, 免疫组织化学, 荧光原位杂交, 组织病理学, Lung neoplasms, *ROS1* gene fusion, Next-generation sequencing, Immunohistochemistry, Fluorescence *in situ* hybridization, Histopathology

## Abstract

**背景与目的** *C-ROS*原癌基因1-受体酪氨酸激酶（*c-ROS *proto-oncogene 1 receptor tyrosine kinase, *ROS1*）基因融合阳性的非小细胞肺癌（non-small cell lung cancer, NSCLC）是肺癌中的高危类型，精准识别这一特殊类型十分关键。本研究旨在通过分析下一代测序（next-generation sequencing, NGS）与免疫组织化学（immunohistochemistry, IHC）及荧光原位杂交（fluorescence *in situ* hybridization, FISH）检测结果的一致性，来验证IHC作为ROS1融合初筛工具的临床价值。**方法** 收集2020年11月1日至2022年11月30日中国医学科学院肿瘤医院收治的NSCLC病例，经NGS检测所有病例*ROS1*基因融合，苏木精-伊红（hematoxylin and eosin, HE）染色进行复核，IHC和FISH检测ROS1蛋白表达和基因断裂，并分析临床病理特征及3种检测方法结果的一致性。**结果** 770例NSCLC共检出22例*ROS1*融合阳性病例。组织学均为肺腺癌，以低分化为主，主要生长模式包括实性型、腺泡型及乳头型等。IHC检测显示22例均呈ROS1蛋白阳性表达，阳性符合率为100.0%，其中14例（63.6%）弥漫性强阳性。FISH检测16例为典型的分离信号模式，阳性符合率为72.7%。NGS、IHC与FISH这3种方法的总符合率为72.7%。**结论** *ROS1*融合阳性NSCLC具有特征性临床病理学特征。在经NGS确认的*ROS1*融合阳性病例中，IHC表现出100.0%的敏感性，提示其具备作为初筛工具的潜力，但其特异性仍需进一步研究验证，推荐在ROS1 IHC强阳性病例中优先采用NGS检测。当IHC强阳性而FISH信号不典型时，NGS是确诊并明确融合伴侣及断裂点的关键技术。联合应用组织形态学评估、IHC初筛与NGS验证的综合诊断策略，有助于实现*ROS1*阳性肺癌的精准诊断，从而指导临床靶向治疗。

## Abstract (en)

**Background and objective** *C-ROS* proto-oncogene 1 receptor tyrosine kinase (*ROS1*) gene fusion-positive non-small cell lung cancer (NSCLC) is a high-risk subtype of lung cancer, and the accurate identification of this specific subtype is critically important. This study systematically analyzes the concordance between the results of next-generation sequencing (NGS), immunohistochemistry (IHC), and fluorescence *in situ* hybridization (FISH) to validate the clinical value of IHC as an initial screening tool for *ROS1* fusions. **Methods** Patients with NSCLC diagnosed at Cancer Hospital, Chinese Academy of Medical Sciences between November 1, 2020 and November 30, 2022 were enrolled. All cases were tested for *ROS1* gene fusion via NGS, re-examined with hematoxylin-eosin (HE) staining, and ROS1 protein expression and gene breakage were detected using IHC and FISH. The results of the three detection methods and their correlation with clinicopathological characteristics were compared. **Results** 22 were detected as *ROS1* fusion-positive among 770 NSCLC cases. Histologically, all were lung adenocarcinoma, predominantly poorly differentiated. The main growth patterns included solid, acinar, and papillary types. IHC detection showed positive expression of ROS1 protein in all 22 cases, including 14 cases (63.6%) with strong diffuse positivity, resulting in a positive concordance rate of 100.0%. FISH detection revealed a typical isolated signal pattern in 16 cases, with a positive concordance rate of 72.7%. The overall concordance rate among NGS, IHC, and FISH was 72.7%. **Conclusion** *ROS1* fusion-positive NSCLC exhibits characteristic clinicopathological features. In *ROS1 *fusion-positive cases confirmed by NGS, IHC serves as a highly sensitive tool with 100.0% sensitivity, suggesting its potential as an initial screening method, but its specificity still needs further verification. NGS testing is recommended as a priority in cases with strong ROS1 IHC positivity. When strong IHC positivity is observed alongside atypical FISH signals, NGS becomes a key technique for confirmation and precise identification of fusion partners and breakpoints. An integrated diagnostic strategy combining histomorphological evaluation, IHC preliminary screening, and NGS validation facilitates accurate diagnosis of *ROS1*-positive lung cancer, thereby guiding clinical targeted therapy implementation.

肺癌是全球范围内发病率和死亡率最高的恶性肿瘤^[1]^，而非小细胞肺癌（non-small cell lung cancer, NSCLC）约占肺癌发病率的85%。近年来，低剂量计算机断层扫描（computed tomography, CT）筛查的普及与分子靶向治疗的广泛应用，共同推动了NSCLC治疗格局的深刻变革。除经典的间变性淋巴瘤激酶（anap1astic lymphoma kinase, ALK）融合外，c-ROS原癌基因1-受体酪氨酸激酶（c-ROS proto-oncogene 1 receptor tyrosine kinase, ROS1）基因融合作为另一个重要的驱动基因（发生率为1%-2%）在NSCLC的靶向治疗中具有重要意义^[2]^。这类患者往往在初诊时就已经出现脑转移（30%-50%）^[3,4]^，预后更差。而新一代的酪氨酸激酶抑制剂（tyrosine kinase inhibitors, TKIs）如Taletrectinib、Repotrectinib等，能突破血脑屏障，增强中枢神经系统渗透性和脱靶神经毒性^[4]^，表现出较高的客观缓解率。因此，高效、准确地识别ROS1阳性患者已成为临床病理诊断的迫切需求。

目前，ROS1基因融合在检测方法的有效性和一致性方面仍存在明显的研究空白。免疫组织化学（immunohistochemistry, IHC）成本低、时间周期短、易于开展，但其特异性低和判读标准无明确共识^[5]^。荧光原位杂交（fluorescence in situ hybridization, FISH）曾被视作“金标准”，但不能多重检测和鉴定ROS1融合伴侣，且操作复杂、判读主观、成本高昂^[2]^。而下一代测序（next-generation sequencing, NGS）是临床常规使用的检测手段，直接检测功能性融合^[6,7]^，但NGS的实验室要求高、操作复杂且成本高昂，因此很多基层医院的病理科无法开展检测工作，导致不能高效准确识别ROS1的基因融合^[8]^；同时，3种检测方法之间的一致性和互补性尚未在临床样本中得到充分验证。此外，现有的研究主要集中在特定的检测技术上，而缺乏对其在临床管理中综合应用的系统性探讨。

本研究旨在弥补上述不足，通过对中国NSCLC患者中ROS1基因融合的流行病学、临床病理特征及分子特征进行系统性描述，并探索不同检测方法的有效性。通过对回顾性队列进行研究分析，系统比较IHC、FISH和NGS这3种检测技术在ROS1融合检测中的表现，以评估IHC作为初筛工具的潜力。研究的特点在于在既往研究基础上，通过应用大规模临床样本对这3种方法进行平行应用与比较，旨在为临床实践提供更为经济高效的检测流程。通过评估不同检测方法，期望为NSCLC患者的精准诊断和个体化治疗提供可靠的依据，从而确保更多患者能够从靶向治疗中获益。

## 1 资料与方法

### 1.1 研究对象

收集2020年11月1日至2022年11月30日中国医学科学院肿瘤医院收治的770例NSCLC患者组织样本，采用NGS平台检测ROS1基因融合变异，其中ROS1融合基因阳性患者的组织样本同步采用IHC检测ROS1蛋白表达、FISH检测ROS1断裂。样本类型包括肺原发肿瘤的手术标本、转移瘤的穿刺标本和淋巴穿刺标本。确定为ROS1基因融合阳性的NSCLC患者存档石蜡标本及临床资料。本研究经北京协和医学院/中国医学科学院肿瘤医院伦理委员会审查批准（审批号：23/095-3844）并经患者签署知情同意。

### 1.2 研究方法

#### 1.2.1 苏木精-伊红（hematoxylin and eosin, HE）染色

组织标本经病理医师取材后至4%多聚甲醛固定过夜，随后全自动脱水机脱水、浸蜡、包埋，将石蜡包埋组织切成4 µm厚的切片，行HE染色，并用光学显微镜观察各组之间的差异。所有HE染色切片由2名资深病理医师采用双盲法进行独立复查。评估肿瘤的组织学亚型[根据第5版世界卫生组织（World Heahth Organization, WHO）肺肿瘤分类]、生长模式、细胞形态特征（如是否存在印戒细胞、黏液分化等）。

#### 1.2.2 增强免疫组织化学（Ventana immunohistochemistry, Ventana IHC）检测方法及判读

本次实验IHC采用Roche公司BenchMark ULTRA全自动免疫组化仪检测肿瘤组织样本中ROS1蛋白的表达。常规石蜡包埋组织，使用切片机连续切片（厚度4 μm），贴附于防脱载玻片，经充分烤片后，将切片置于全自动免疫组化仪的相应位置，并将一抗（ROS1兔单克隆抗体，克隆号VENTANA SP384）、OptiView DAB增强检测试剂盒（Roche公司）、OptiView扩增检测试剂盒（Roche公司）、苏木精染液及返蓝液置于试剂架上，启动检测程序，完成上机操作。以已知阴性病例作为阴性对照。染色完成后进行脱水、透明及封片处理，于显微镜下观察结果。结果判定标准如下。IHC 0（阴性）：瘤细胞胞浆无明显着色或≤10%胞浆微弱棕色着色；IHC 1+：>10%的肿瘤细胞胞浆呈现微弱棕色着色，且无任何背景染色；IHC 2+：>10%的肿瘤细胞胞浆呈现棕色着色；IHC 3+：>10%的肿瘤细胞胞浆呈现深棕色强着色。本实验所有HE及IHC判读结果均经病理科2位高年资病理医生按照结果判定要求进行双盲判读，如诊断意见不一致由双方于多头镜共同判断达成一致意见。未采用自动图像分析系统。

#### 1.2.3 FISH检测方法及判读

ROS1基因重排的检测选用双色分离探针。ROS1基因5'端标记为红色，长度为220 kb（chr6: 117,658,894-118,064,025），ROS1基因3'端标记为绿色，长度为460 kb（chr6: 116,998,724-117,599,018），实验严格按照ROS1基因断裂重组检测试剂盒（绍兴金箓生物技术有限公司）说明书进行操作，染色后使用徕卡DM6000荧光显微镜分析荧光信号。由经过培训的病理技术人员和病理诊断医师对每份标本的至少100个非重叠肿瘤细胞核进行评分。FISH结果判读标准如下：（1）以下情况细胞为阴性（非重排的）：①红色和绿色信号相邻或重合（在红/绿双通滤光片下观察为黄色），红绿信号分离，但距离≤两个信号直径的，这种也视为融合信号，②单独存在红色信号，不伴有对应绿色信号；（2）以下情况细胞为阳性（发生重排的）：①至少有1对红色和绿色信号分离的距离超过2个信号直径的，②除了融合和/或分离信号外，单独存在绿色信号，不伴有对应红色信号，③如果阳性细胞占计数肿瘤细胞的比例≥15%，判为ROS1基因存在断裂重排。

#### 1.2.4 NGS

首先，对样本进行总DNA提取，随后对DNA进行定量和片段分析达到质控要求。随后通过DNA片段化、末端修复、接头连接与片段筛选等步骤构建文库。之后利用探针杂交捕获文库，并对文库进行聚合酶链式反应（polymerase chain reaction, PCR）扩增。使用Illumina测序仪对文库进行双端测序以实现高通量测序。测序原始数据经过滤后，进行生物信息学分析，以GRCh37/hg19为参考基因组序列，从比对结果中同步分析基因点突变、插入缺失、基因拷贝数变异和融合变异等多种突变类型。其中，NGS panel覆盖ROS1基因的全外显子及部分内含子区域，不仅限于重排位点位于第33、34、35内含子区的融合，还包括G2032R耐药位点等突变以及拷贝数变异。肿瘤组织的平均测序深度为1000×。

### 1.3 统计学分析

采用SPSS 26.0软件进行数据分析。计数资料以率（%）表示，组间比较采用χ²检验或Fisher精确检验。连续性变量如不符合正态分布采用中位数（范围）描述，组间比较采用Mann-Whitney U检验。采用Kappa一致性检验评估两两检测方法（NGS与IHC、NGS与FISH）之间的一致性，Kappa值≥0.75为一致性良好，0.40-0.75为一致性中等，<0.40为一致性较差。以P<0.05为差异具有统计学意义。

## 2 结果

### 2.1 患者临床特征

770例NSCLC患者组织样本采用NGS平台检测ROS1融合基因变异，其中22例ROS1融合阳性NSCLC患者的组织样本同时采用IHC检测ROS1蛋白表达、FISH检测ROS1断裂。共纳入22例ROS1融合阳性NSCLC患者（4例为淋巴结转移穿刺病例），其中男性9例，女性13例；患者年龄29-85岁，中位年龄为53岁。从不吸烟者18例（81.8%），轻度吸烟者（≤10包年）2例（9.1%），中度及以上吸烟者（>10包年）2例（9.1%）。有18例患者标本进行病理肿瘤原发灶-淋巴结-转移（pathological tumor-node-metastasis, pTNM）分期，其中T1期为8例（包括T1a、T1b、T1c），T2期为8例（包括T2a、T2N），T3期为2例；N0期为10例，N1期为2例，N2期为6例。与ROS1融合阴性组相比，阳性组患者年龄更小（中位年龄53 vs 61岁，P<0.05），T分期及N分期、吸烟史和分化程度分布差异具有统计学意义（P<0.05）（表1），性别差异无统计学意义。

**表1:** 患者临床病理特征比较

Variable	Total (n=770)	ROS1 fusion-positive (n=22)	ROS1 fusion-negative (n=748)	P
Sex				0.612
Male	302 (39.2%)	9 (40.9%)	293 (39.2%)	
Female	468 (60.8%)	13 (59.1%)	455 (60.8%)	
Age (yrs)				0.042
Median (range)	61 (27-88)	53 (29-85)	61 (27-88)	
Smoking history				0.038
Never smoked	507 (65.8%)	18 (81.8%)	489 (65.4%)	
Light smoking (≤10 pack-years)	100 (13.0%)	2 (9.1%)	98 (13.1%)	
Moderate or heavy smoking (>10 pack-years)	163 (21.2%)	2 (9.1%)	161 (21.5%)	
T stage*				<0.001
T1	490 (79.5%)	8 (44.4%)	482 (80.6%)	
T2	91 (14.8%)	8 (44.4%)	83 (13.9%)	
T3	35 (5.7%)	2 (11.1%)	33 (5.5%)	
T4	0 (0.0%)	0 (0.0%)	0 (0.0%)	
N stage*				<0.001
N0	540 (87.7%)	10 (55.6%)	530 (88.6%)	
N1	28 (4.5%)	2 (11.1%)	26 (4.3%)	
N2	48 (7.8%)	6 (33.3%)	42 (7.0%)	
Histological differentiation				<0.001
Well differentiation	68 (8.8%)	0 (0.0%)	68 (9.1%)	
Moderate differentiation	418 (54.3%)	6 (27.3%)	412 (55.1%)	
Poorly differentiattion	284 (36.9%)	16 (72.7%)	268 (35.8%)	

*T staging and N staging data in the ROS1 fusion positive group were calculated based on 18 cases with available pTNM staging information (rather than all 22 cases). T staging and N staging data in the ROS1 fusion-negative group were calculated based on 598 cases with available pTNM staging information (rather than all 748 cases), as only 598 of 748 patients had pTNM staging records. ROS1: c-ROS proto-oncogene 1 receptor tyrosine kinase; pTNM: pathological tumor-node-metastasis.

### 2.2 NGS检测结果

NGS检测阳性率2.9%（22/770），其中CD74::ROS1融合变异11例，SDC4::ROS1、TPM3::ROS1、EZR::ROS1融合变异各2例，AFG1L::ROS1、CCDC6::ROS1、SLC34A2::ROS1、SNX25::ROS1、LRRK2::ROS1融合变异1例（表2和图1A）。在22例ROS1融合阳性病例中，未检测到表皮生长因子受体（epidermal growth factor receptor, EGFR）、Kirsten大鼠肉瘤病毒癌基因同源物（Kirsten rat sarcoma viral oncogene homolog, KRAS）、B-Raf原癌基因（B-Raf proto-oncogene, BRAF）等其他驱动基因突变共存。

**表2:** IHC和FISH检测经NGS测得的ROS1已知融合突变结果对比

No.	Sex	Age (yrs)	Smoking status	Histological type	pTNM stage	ROS1-NGS	ROS1-FISH	ROS1-IHC
1	Male	70	Moderate or heavy smoking	Moderately differentiated	pT1aN0	CD74::ROS1	Positive	3+
2	Female	62	Never smoked	Poorly differentiated	pT3N0	TPM3::ROS1	Atypical	2+
3	Male	36	Never smoked	Poorly differentiated	ypT2aN0	CD74::ROS1	Positive	2+
4	Female	64	Never smoked	Poorly differentiated	pT1cN0	EZR::ROS1	Atypical	2+
5	Female	40	Never smoked	Poorly differentiated	-	CD74::ROS1	Atypical	3+
6	Female	38	Never smoked	Poorly differentiated	-	CD74::ROS1	Positive	3+
7	Female	46	Never smoked	Poorly differentiated	pT2aN2	CD74::ROS1	Positive	3+
8	Male	60	Light smoking	Poorly differentiated	pT2aN0	CD74::ROS1	Positive	3+
9	Female	72	Never smoked	Poorly differentiated	-	SLC34A2::ROS1	Atypical (weak signal, unable to determine)	1+
10	Female	67	Light smoking	Moderately to poorly differentiated	pT1cN0	SDC4::ROS1	Positive	3+
11	Female	34	Never smoked	Moderately differentiated	pT3N0	CD74::ROS1	Positive	3+
12	Female	45	Never smoked	Moderately differentiated	pT1bN1	LRRK2::ROS1	Positive	3+
13	Male	60	Never smoked	Moderately differentiated	pT2aN0	AFG1L::ROS1	Positive	3+
14	Male	72	Never smoked	Poorly differentiated	pT2N0	TPM3::ROS1	Positive	2+
15	Female	39	Never smoked	Poorly differentiated	pT1cN2	SNX25::ROS1	Positive	3+
16	Male	74	Never smoked	Poorly differentiated	pT2aN1	CCDC6::ROS1	Positive	1+
17	Male	47	Never smoked	Poorly differentiated	pT2N2	CD74::ROS1	Positive	1+
18	Male	62	Never smoked	Moderately to poorly differentiated	pT1cN2	CD74::ROS1	Positive	3+
19	Female	65	Moderate or heavy smoking	Poorly differentiated	-	CD74::ROS1	Atypical	3+
20	Female	41	Never smoked	Poorly differentiated	pT1bN0	SDC4::ROS1	Atypical	3+
21	Female	37	Never smoked	Poorly differentiated	pT2N2	EZR::ROS1	Positive	2+
22	Male	44	Never smoked	Poorly differentiated	pT1bN2	CD74::ROS1	Positive	3+

The "FISH atypical" pattern manifests as 1F1G "1 fusion signal with 1 green signal" mode (Caused by the absence of the 5′-end red probe signal and retention of the 3′-end green probe signal). IHC: immunohistochemistry; FISH: fluorescence in situ hybridization; NGS: next-generation sequencing; CD74: cluster of differentiation 74; SDC4: syndecan 4; TPM3: tropomyosin 3; EZR: ezrin; AFG1L: ATPase; CCDC6: coiled-coil domain containing 6; SLC34A2: solute carrier family 34 member 2; SNX25: sorting nexin 25; LRRK2: leucine rich repeat kinase 2.

**图1:**
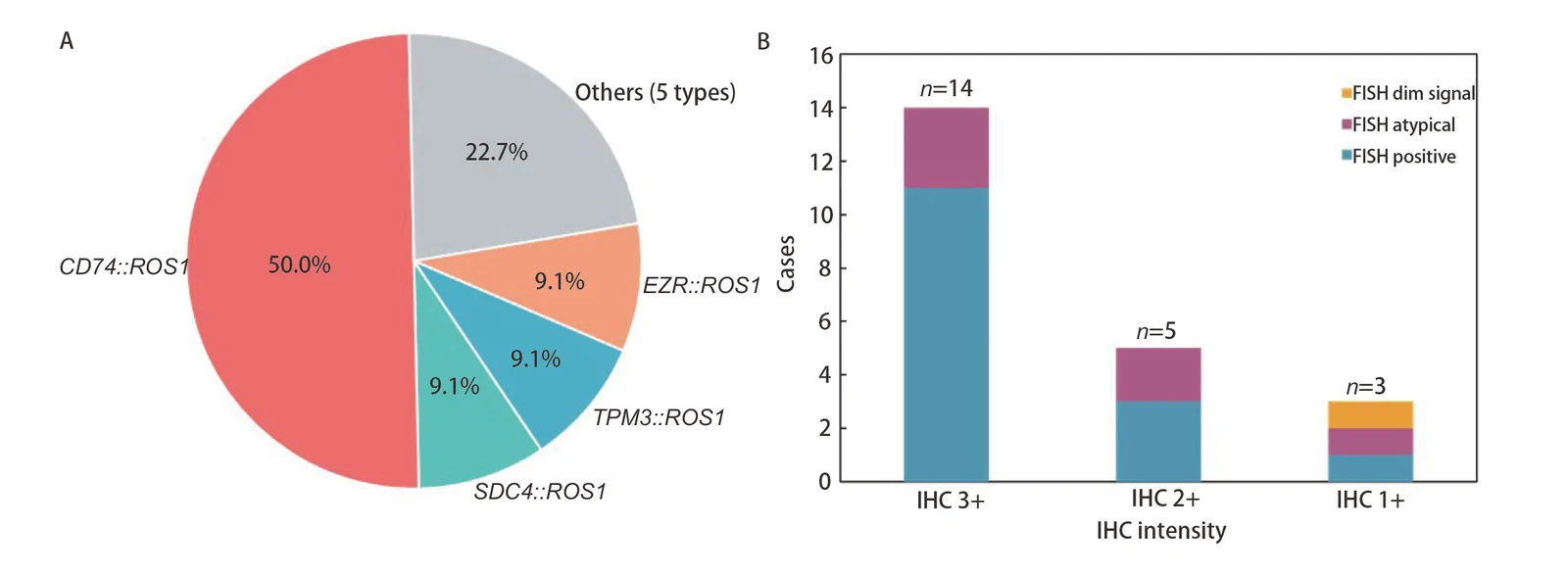
ROS1融合阳性非小细胞肺癌分析（n=22）。A：ROS1融合伙伴的分布；B：IHC强度与FISH模式的关联。

### 2.3 组织病理学结果

22例组织学均为肺腺癌，其中低分化腺癌16例（72.7%），中分化腺癌4例（18.2%），中-低分化腺癌2例（9.1%）。主要的组织学亚型分布为：实体型（图2A）、筛状结构（实性型、腺泡型）（图2B）、乳头型及微乳头型（图2C）等。其中，1例可见印戒样细胞，即胞质内黏液产生（图2D）。

**图2:**
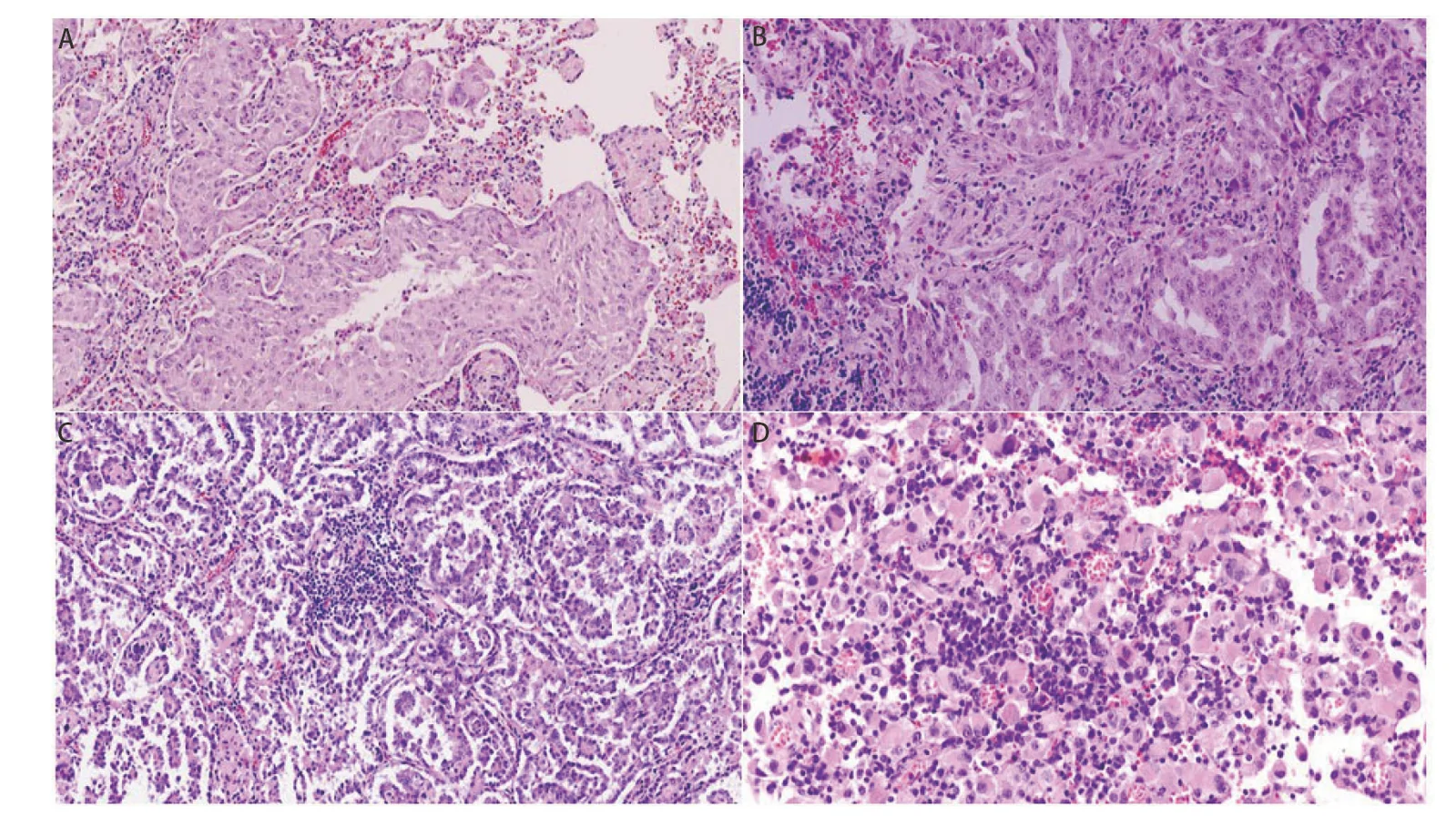
ROS1阳性肺腺癌的病理学HE染色结果。A：组织学亚型为实性型（×400）；B：组织学亚型为筛状结构（×400）；C：组织学亚型为乳头型及微乳头型（×400）；D：印戒样细胞（×400）。

### 2.4 IHC结果

所有经NGS确诊为ROS1融合的病例，其IHC检测均显示ROS1蛋白阳性表达（表2和图1B）。阳性着色强度具体分布为：1+阳性3例，2+阳性5例（图3A-3C），3+强阳性14例（占63.6%，图3D）。

**图3:**
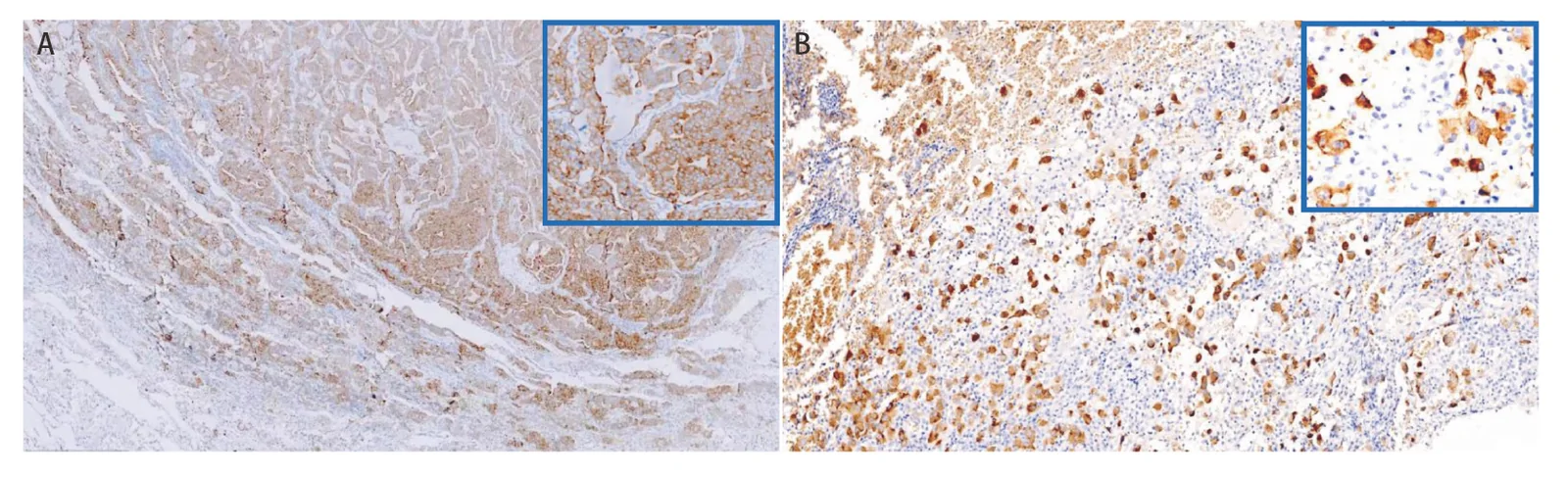
ROS1 IHC染色结果。A：ROS1蛋白在肿瘤细胞胞浆呈2+阳性表达（×200）；B：ROS1蛋白在肿瘤细胞胞浆呈3+阳性表达（×200）。右上角放大部分的倍数为×400。

### 2.5 FISH检测结果

在22例病例中16例（72.7%）显示典型的分离信号模式，判读为FISH阳性（表2）。另有5例（22.7%）表现为不典型信号模式，主要为“单一绿色信号”（即仅见3’端绿色信号，5’端红色信号缺失，提示可能存在5’端探针结合区域的缺失，导致分离信号未呈现典型模式。因不典型信号的分子机制为推测性解释，本研究未提供直接分子证据如反转录PCR（reverse transcription-PCR, RT-PCR）验证，存在证据链不足的局限性。1例（4.6%）因信号较弱无法明确判读。FISH不典型信号病例对应的融合伴侣分别为TPM3::ROS1（1例）、EZR::ROS1（1例）、CD74::ROS1（2例）、SDC4::ROS1（1例）（图4A）。

**图4:**
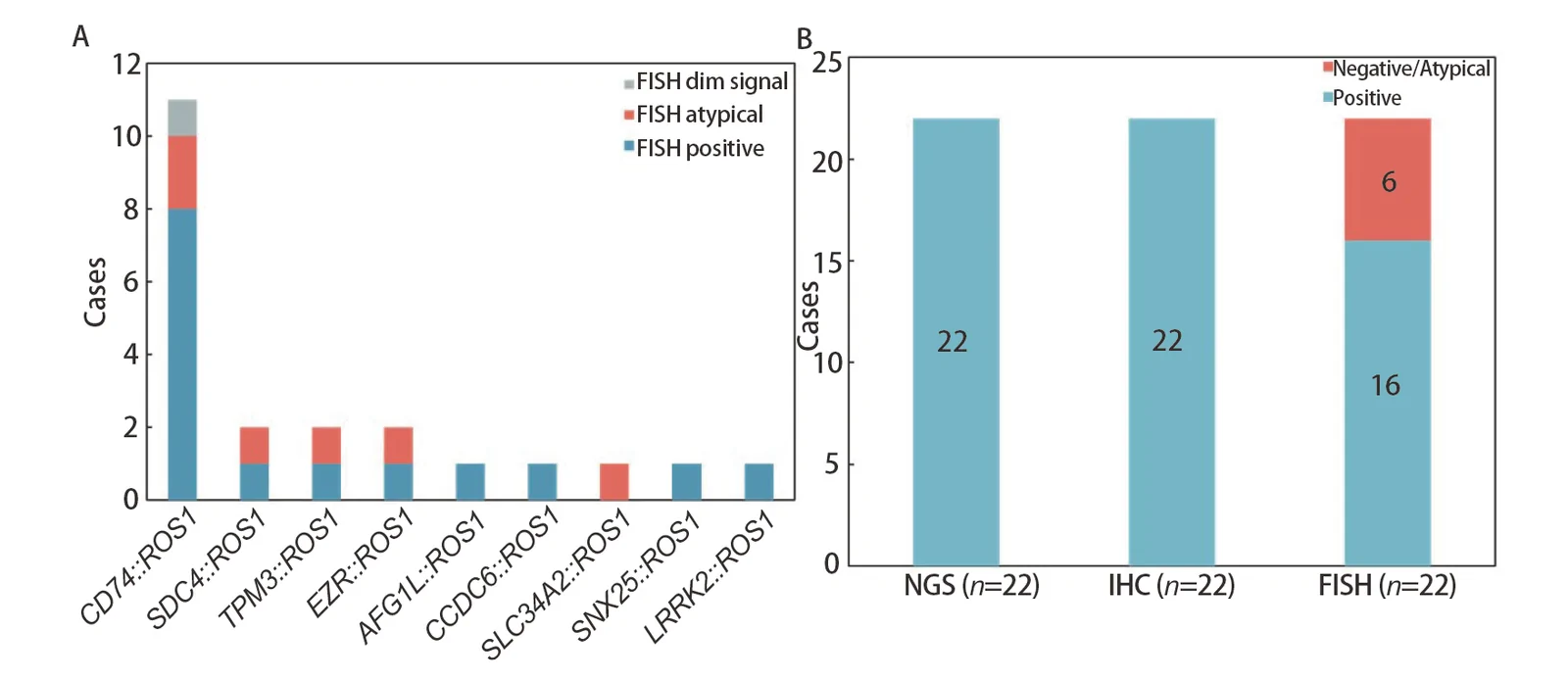
按融合伴侣分组的FISH信号模式与方法一致性分析。A：按融合伴侣划分的FISH信号模式；B：3种检测方法的一致性。

### 2.6 检测方法间的一致性分析

NGS与IHC的阳性符合率为100.0%（Kappa=1.000）（图4B）。NGS与FISH（以典型分离信号为标准）的阳性符合率为72.7%（16/22），因NGS均为阳性，无法计算有意义的Kappa值（图4B）。在NGS与IHC均为阳性的前提下，3种方法结果完全一致的病例占72.7%（16/22）。吸烟史与融合亚型（CD74::ROS1融合与非CD74::ROS1融合）无显著关联（Fisher精确检验，P=0.293），比值比为3.75（95%CI: 0.34-41.84）。IHC强度与FISH信号模式无显著关联（P=0.396）。在所有NGS鉴定出的融合伴侣基因中，CD74为最常见的融合伴侣，其次为SDC4、TPM3、EZR等。

## 3 讨论

本研究围绕ROS1阳性NSCLC的临床病理特征及3种检测方法的应用价值展开，核心发现如下：（1）在770例NSCLC患者中，ROS1融合阳性率为2.9%，其中CD74为最常见的融合伴侣；（2）IHC检测在本研究队列中表现出极高的敏感性（100.0%），所有经NGS确诊的ROS1融合阳性病例均呈现ROS1蛋白阳性表达，提示其具备作为ROS1融合初筛工具的良好潜力；（3）FISH检测存在一定局限性，仅72.7%的阳性病例表现为典型分离信号模式，另有27.3%的病例呈现不典型信号模式，提示其在特定情况下可能存在漏检风险。基于上述核心发现，本研究进一步探讨ROS1融合阳性NSCLC的临床病理特征、3种检测方法的优劣及临床应用价值，为临床精准诊断提供参考。

NSCLC是最常见的肺癌类型，占所有肺癌病例的85%^[9]^。尽管近年来NSCLC的治疗取得了显著进展，但其高发病率和死亡率依旧对全球公共卫生构成重大威胁^[10]^。ROS1基因融合作为一种较为罕见的驱动基因变异，在NSCLC的靶向治疗中具有重要意义，因此准确检测ROS1融合对实现精准治疗至关重要^[11]^。本研究针对中国人群中的NSCLC患者，系统性比较了3种ROS1融合检测方法，即NGS、IHC和FISH。研究发现，在770例NSCLC患者中，ROS1融合的阳性率为2.9%，CD74为最常见的融合伴侣。所有NGS确认的ROS1融合病例均在IHC中表现出阳性表达，且NGS与FISH检测的符合率为72.7%。这些结果为IHC在ROS1融合初筛中的应用潜力提供了重要支持，强调了多平台检测在优化临床管理路径中的重要性。

本研究在770例中国NSCLC患者中检测到ROS1融合的阳性率为2.9%，该结果与全球其他地区的研究结果存在差异，后者通常报告的发生率为1%-2%^[10]^。这一差异可能归因于人群基因组特征、样本选择及检测技术的灵敏度不同^[10,11]^。例如，本研究中CD74作为最常见的融合伴侣，可能与该人群的遗传背景和环境因素有关^[7]^。与其他伴侣（如SDC4、TPM3）相比，CD74可能在肿瘤的致癌机制、对TKIs的反应以及预后方面存在显著差异^[12,13]^。这些发现为进一步研究ROS1融合伴侣的生物学功能和临床价值提供了重要依据。

本研究中，22例ROS1融合阳性患者的临床特征与既往文献报道高度一致，即多见于无吸烟史或轻度吸烟的年轻女性，病理类型全部为腺癌^[3]^，且以低分化腺癌为主，这与EGFR和ALK等其他驱动基因阳性肺癌的典型病理特征存在显著差异^[14]^。上述结果表明，ROS1融合阳性肺腺癌可能具有与其他驱动基因阳性肺癌不同的临床行为模式，从而在病理识别和诊断时需要特别警觉^[14]^，当病理医师镜下观察到具有特定组织学亚型（如实性型或微乳头型）的肺腺癌，尤其是年轻非吸烟患者时应高度警惕存在ROS1融合的可能性，从而开展针对性的分子检测。

本研究显示，IHC在本队列中展现出卓越的敏感性（100.0%），所有经NGS确认的阳性病例均呈现ROS1蛋白表达。其中，超过半数的病例为弥漫性强阳性（3+），这与致癌性融合驱动导致ROS1激酶结构域持续激活并过表达的理论完全相符。该结果进一步支持了ROS1 IHC作为高效、经济初筛工具的应用价值^[15]^，尤其适用于条件有限的医疗机构，可有效缩小后续进行分子检测的病例范围。但本研究未纳入IHC阳性而NGS阴性的病例，无法评估IHC的特异性、假阳性率及阳性预测值，既往研究^[9]^表明ROS1 IHC阳性病例中仅有少数经FISH或分子方法证实为真正的ROS1融合，提示IHC存在一定的假阳性率，其临床应用的可行性有待前瞻性、连续入组的大样本研究进一步验证。

然而，与IHC的高敏感性不同，FISH检测结果呈现出显著的复杂性。本研究中，仅72.7%病例表现为经典的“分离信号”模式。这种现象很可能源于特定的基因断裂事件：结合本研究使用的双色分离探针设计（5’端红色，3’端绿色），当断裂点位于ROS1基因的3’端探针结合区域内时，会导致5’端序列缺失，使得红色标记的探针无法结合，从而仅留下3’端绿色探针的信号，形成“单一绿色信号”的不典型模式^[16]^。这一发现具有重要的临床提示意义：若严格遵循传统的“分离信号”判读标准，可能导致部分（本研究中近30%）ROS1阳性病例被漏诊。这表明，曾被视为“金标准”的FISH技术，其检测效能高度依赖于探针的设计及判读人员对不典型模式的认知与经验。在面对特定基因如ROS1时，其“金标准”地位已受到严峻挑战。需要说明的是，本研究中对FISH不典型信号的机制解释为基于探针设计和现有文献的理论推测，未采用RT-PCR等技术进行交叉验证，存在证据链不足的问题，这也是本研究的局限性之一。

在此情况下，NGS的技术优势则更为突出^[5]^。NGS不仅独立于探针结合位点直接检测到基因融合，更重要的是，它能精准鉴定融合伴侣基因^[17]^。本研究中除了检出最常见的CD74::ROS1（占50.0%）外，还识别出包括SDC4、TPM3、EZR在内的多种罕见伴侣类型^[18]^。随着更多靶向药物的研发与应用，不同融合伴侣可能对TKIs药物的疗效产生影响已初见端倪^[19]^。因此，NGS所提供的完整基因信息，对于指导个性化的靶向治疗及预测耐药机制具有不可替代的价值。当IHC初筛为强阳性而FISH结果为不典型模式时，NGS成为了最终确诊并提供全面基因组信息的决定性方法。

基于此，本研究建议采用一种多平台、分层次的整合诊断流程，以期在保证诊断准确性的同时，兼顾效率与成本效益。对所有肺腺癌，或具备高危临床/形态学特征（年轻、非吸烟、实体型伴印戒细胞等）的病例，常规进行ROS1 IHC检测。对IHC弥漫性强阳性（2+/3+）的病例，优先采用NGS进行确认，并一次性获取融合伴侣、共突变等全面信息。在IHC与NGS结果不符或无法进行NGS检测时，可使用FISH进行验证，但判读标准必须纳入对“单一红色/绿色信号”等不典型模式的识别。

本研究存在若干局限性：（1）研究中仅纳入22例ROS1融合阳性病例，样本量相对较小，可能影响统计分析的效力，并限制了对罕见融合伴侣及特殊临床病理亚型的深入探讨；（2）由于采用回顾性研究设计，可能存在选择偏倚，同时未能获取所有病例的完整随访和治疗反应数据，限制了对预后及疗效的分析；（3）本研究只展现了22例NGS阳性的ROS1 IHC分析结果但未纳入IHC阳性而NGS阴性的病例队列，未展现“IHC特异性”“假阳性率”及“阳性预测值”这些指标，其临床应用的可行性有待在前瞻性、连续入组的大样本研究中进一步验证；（4）罕见融合伴侣（如LRRK2::ROS1、SNX25::ROS1）仅各1例，样本量不足，无法开展统计分析，其临床意义需大样本研究验证；（5）缺乏患者的生存数据使得本研究无法直接评估ROS1作为生物标志物的预后和预测价值；（6）对FISH不典型信号的分子机制仅为理论推测，缺乏RT-PCR交叉验证，证据链不足。未来需要开展更大规模、前瞻性的多中心研究以验证本研究结论，并深入探索不同融合伴侣对各类TKIs药物（如Taletrectinib、Repotrectinib）治疗反应的潜在影响^[20,21]^，这将是迈向更精准医疗的关键一步。

本研究证实，ROS1融合阳性NSCLC是一类具有独特临床病理特征的分子亚型。在经NGS确认的ROS1融合阳性病例中，IHC表现出100.0%的敏感性，提示其具备作为初筛工具的潜力，但其特异性仍需进一步研究验证，推荐在ROS1 IHC强阳性病例中优先采用NGS检测。面对FISH不典型信号时，NGS是最终确诊和获取全面基因组信息的决定性方法。综合组织学形态评估、IHC初筛与NGS验证的诊断策略，有助于确保ROS1阳性患者不被漏诊，从而及时从精准靶向治疗中获益。
